# Computational gastronomy: capturing culinary creativity by making food computable

**DOI:** 10.1038/s41540-024-00399-5

**Published:** 2024-07-08

**Authors:** Ganesh Bagler, Mansi Goel

**Affiliations:** 1 Department of Computational Biology, Indraprastha Institute of Information Technology Delhi (IIIT-Delhi), Okhla Phase III, New Delhi, 110020 India; 2 Infosys Center for Artificial Intelligence, Indraprastha Institute of Information Technology Delhi (IIIT-Delhi), Okhla Phase III, New Delhi, 110020 India; 3 Center of Excellence in Healthcare, Indraprastha Institute of Information Technology Delhi (IIIT-Delhi), Okhla Phase III, New Delhi, 110020 India

**Keywords:** Computational biology and bioinformatics, Systems biology, Computer science

## Abstract

Cooking, a quintessential creative pursuit, holds profound significance for individuals, communities, and civilizations. Food and cooking transcend mere sensory pleasure to influence nutrition and public health outcomes. Inextricably linked to culinary and cultural heritage, food systems play a pivotal role in sustainability and the survival of life on our planet. Computational Gastronomy is a novel approach for investigating food through a data-driven paradigm. It offers a systematic, rule-based understanding of culinary arts by scrutinizing recipes for taste, nutritional value, health implications, and environmental sustainability. Probing the art of cooking through the lens of computation will open up a new realm of possibilities for culinary creativity. Amidst the ongoing quest for imitating creativity through artificial intelligence, an interesting question would be, ‘Can a machine think like a Chef?’ Capturing the experience and creativity of a chef in an AI algorithm presents an exciting opportunity for generating a galaxy of hitherto unseen recipes with desirable culinary, flavor, nutrition, health, and carbon footprint profiles.

## Cooking as an art

Cooking is the art of creatively combining and processing natural ingredients to arrive at delicious dishes magically^1^. Humans are unique in having evolved the art of cooking^2^. Over millennia, this creative process has led to rich culinary legacies that define cultures worldwide^1,3,4^. Similar to languages that embody linguistic histories, cuisines capture the culinary legacies of cultures. Can we leverage the rich culinary knowledge to create a rule-based understanding beyond the artistic outlook towards cooking? Imagine an AI-generated recipe winning the MasterChef show! While it may seem preposterous, algorithmic protocols that mimic cognitive and sensory processes may soon embrace cooking, similar to board games (such as Chess and Go), literature, art, and music.

Centuries ago, inventing microscopes enabled us to look deeper to probe cellular mysteries and build better models of living things. Today, data is the new microscope. The application of data- and computing-driven approaches has demonstrated the utility of data science in various endeavors, ranging from playing chess to predicting the weather. Can we combine the jigsaw pieces of the food puzzle to leverage data science’s power for better nutrition, health, and a sustainable food system? Do we have data on all relevant aspects of food and cooking?

Traditionally, gastronomy is seen from an artistic perspective^5^. Matters ranging from pleasure to cultural nuances and cooking to health associations have been primarily approached qualitatively. However, the increasing availability of structured data and the advent of computational methods are dramatically changing the artistic outlook toward gastronomy. The application of data-driven strategies for investigating gastronomic questions has opened up an all-new paradigm for the study of food and cooking. Computational Gastronomy is a data science that blends food, data, and computation towards achieving data-driven food innovations^6^. A data and computing-centric approach will enable the transformation of the food landscape leading to better public health and nutrition.

**Fig. 1:**
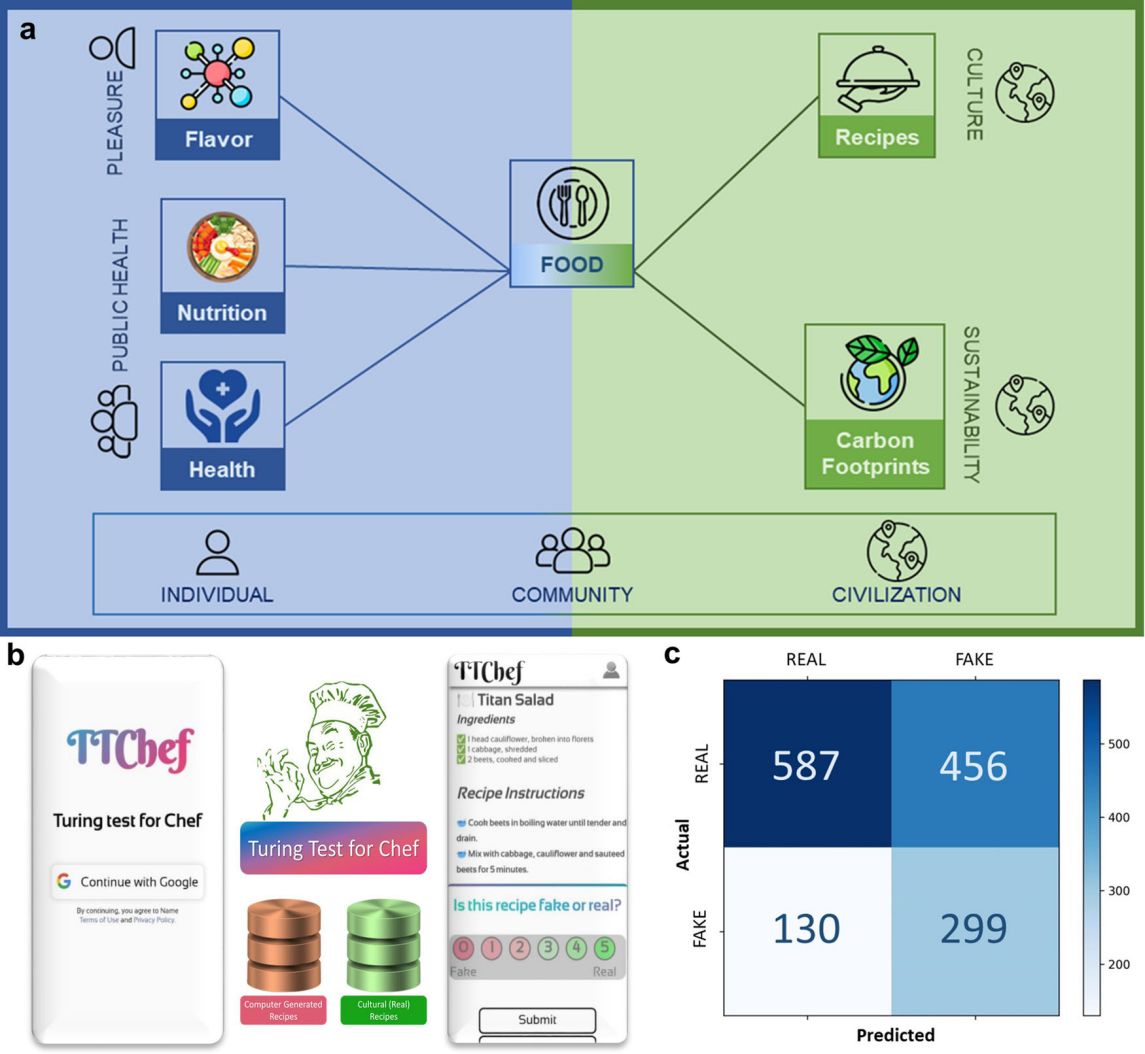
Capturing culinary creativity with computational gastronomy. **a** Making food computable by seeing it through the lens of flavor, recipes, nutrition, health, and sustainability. **b** ‘Turing Test for Chef’ framework for judging the efficacy of computer-generated recipes in fooling a chef. Chefs present their assessment of the recipe text on a scale of 0–5 (Fake—Real) after randomly selected recipes are presented to them from stacks of authentic (real) and computer-generated (fake) recipes. **c** The ‘confusion matrix’ presents the preliminary results of the Turing Test for Chef for recipes generated using a version of Ratatouille algorithm^21^ implementing GPT2 as the underlying text generation model on which the fine-tuning was performed using the data of over 118,000 RecipeDB recipes from across the global cuisines.

In the last nine years, our lab (Complex Systems Laboratory, IIIT-Delhi: https://cosylab.iiitd.edu.in) has built databases^7–11^ and created a body of knowledge^6–31^ that has contributed to the growth of Computational Gastronomy–the data science of food, flavors, nutrition, health, and sustainability (Fig. 1a). Over time, there has been increasing interest in this niche from academia and industry to ask deep gastronomic questions, harvest culinary data, and build algorithms. From food pairing analysis^29,31–40^, study of culinary fingerprints^29,41^, cuisine classification^16^, cuisine evolution^12,13,42^, taste prediction^14,23,43^, exploring phenomena in nutritional profiles^44^, building recipe repositories^7,45,46^, named entity recognition in recipes^17,27,47–53^, novel recipes generation^21,54–58^, and dish detection models^22,24^, to prediction of food processing classes^59^, the frontiers of computational gastronomy have been expanding for over a decade of ever-growing research. These studies have enhanced our understanding of the patterns in recipe composition, the way cuisines evolve and their interrelatedness, the quintessential features that characterize cuisines, the molecular correlates of taste, and enabled the application of text generation, computer vision, machine learning, and deep learning for a host of use cases. Computational gastronomy opens up a new realm of possibilities to transform the food ecosystem by asking exciting questions about food and cooking.

Among the major challenges in the growth of this niche is the creation of structured compilations of rich data with cultural, taste, nutritional, health, and sustainability correlates of food. The diversity of dictionary of food ingredients across the geography, variation in their nutritional and flavor profiles, and origin-specific carbon footprints add to the complexity of the problem. The availability of curated, quality data and techniques for their analysis are among other challenges hindering the growth of this niche. Given the broad spectrum of computational gastronomy applications, some of these challenges should be adequately addressed with increasing interest and participation from stakeholders across academia, government, industry, and the startup ecosystem.

## Food as a complex system

Complex systems^60^ comprise a large number of entities intricately intertwined with each other, giving rise to emergent properties. In these systems, the sum of the parts is not the same as the whole. Various aspects of food, such as the recipe compositions^7^, the experience of the flavor^8^, the nutritional outcome^7^, the health consequences arising out of food^9^, and the carbon footprints^10^ of the food system, exhibit complex systems phenomena. The aggregate system response is an emergent property of its constituent elements in each of them.

To begin with, one can simplify each of these facets of food to make certain assumptions. Given the complexity of the phenomena under consideration and the lack of quantitative formalism, this is expected to be the first step that could be elaborated later to provide the necessary details. Accordingly, a recipe may be defined as an unordered list of ingredients. Clearly, a recipe is a cultural capsule laden with subtle nuances, and such a definition, while convenient for analyses, misses out on many critical factors such as the quantity of each ingredient, their sensory potency, the order in which they are used while cooking, the method used for processing, among others. In reality, the rich sensory experience of a dish is an emergent property arising out of the complex interplay between its constituent elements and processes.

Evolution has endowed humans with a sense of taste and odor central to food appreciation^61,62^. The flavor, experienced through the sensory mechanisms, is an emergent property. Flavor sensation arises from a cocktail of molecules interacting with our sensory apparatus. Analogous to the recipe, an ingredient may be defined as an unordered set of its constituent flavor molecules. However, the concentration of flavor molecules and their potency play a vital role in the flavor profile. Beyond that, the synergy among the flavor compounds and transformation while undergoing the cooking also contribute to the final flavor of the ingredient. Data-driven understanding of flavor correlates of ingredients can provide deep insights into the molecular basis of flavor and answers to many sought-after questions: Why do we pair ingredients in our recipes the way we do? How to enhance the palatability of the food?

Among the data resources created to capture culinary correlates, RecipeDB^7^ presents a structured compilation of over 118,000 recipes from 74 countries. The culinary specifics of these recipes (such as recipe name, ingredient names, quantity, unit, processing, cooking protocol, utensils used, and such) were identified using state-of-the-art named entity recognition (NER) algorithms^17,27^. Further, FlavorDB^8^ provides extensive data on flavor profiles of 936 natural ingredients used in cooking protocols.

## Nutrition and health: meal as a medicine

Humans have evolved over millions of years to become one of the most dominant species on Earth. The ability to cook has been argued to be critical to the rise of a disproportionate increase in the *Homo sapien* brain size^2^. Over the course of human evolution, along with enhanced cognitive abilities, humans also developed a lifestyle that predisposes them to diseases such as obesity, type 2 diabetes, and cardiovascular disorders. Diet is an essential factor contributing to the rise of the epidemic of lifestyle disorders beyond genetic predisposition, social circles, and sedentary work profiles. The search for understanding the basis of nutrition has led to a reductionist breakdown of food into its macro- and micro-nutrients^63^.

Further, the scientific evidence for the health impacts of food is full of contradictory assertions. Our inability to associate food with health consequences reflects the emergent properties arising from subtleties of factors associated with food, the human body, and their interactions. A structured compilation of health associations of food can help us wade through the noise to identify evidence-based dietary interventions. Besides presenting potential solutions for lifestyle disorders, a data-driven approach can help mediate nutritional deficiencies central to public health concerns.

SpiceRx^9^ provides extensive data on spice-disease associations for culinary herbs. It also provides data on phytochemicals, thereby presenting a tripartite resource with immense value. DietRx^11^ takes this to the next level to extend the database and includes over 2,200 food ingredients from across 24 categories. It also provides empirically text-mined quadrangular associations among food ingredients, diseases, chemicals, and genes. These data could be investigated for meaningful inferences for dietary interventions and diet design. Most of the empirical data on the health impact of food are available for individual ingredients. However, to be helpful and culturally relevant, such data must logically be assessed for the complete dish.

One of the most exciting directions from data-driven and evidence-based food investigations is ‘personalized nutrition^64,65^.’ In a pioneering study, researchers meticulously collected data on personal features, such as the nature of gut microbes, blood reports, body measures, and food habits, from a large cohort of people. After substituting one of their meals with a standardized diet, these features were correlated with post-meal glucose levels, closely linked to type 2 diabetes, using a machine learning algorithm. Interestingly, such a ‘personalized nutrition predictor’ could accurately predict the expected rise in glucose levels in repeat experiments with new subjects. More importantly, it could also suggest a personalized dietary recommendation to mitigate glucose levels successfully. Such studies are leading the search for personalized nutrition solutions for diet-linked diseases.

Personalized nutrition may sound like science fiction^65^. However, even weather predictions were considered infeasible just a few decades back. Despite the weather being a complex system dominated by non-linear phenomena, the availability of a large amount of climate data and the application of computational and mathematical techniques have transformed meteorology into a believable science today, at least for short-term predictions. The day is not too far when we can identify personalized, diet-based interventions for many lifestyle disorders and leverage food for better health.

## Ratatouille: generating novel recipes

Chefs embody culinary creativity^1^. As custodians and creators of recipes, the subtle cooking protocols passed through generations for making delicious dishes, chefs preserve the traditional recipes and create new ones. Does the act of cooking have an underlying structure? If yes, can the same be captured through an algorithmic protocol?

Recipes are structured texts involving culinary named entities^25^. Leveraging the power of large language models to generate novel recipes has immense applications for culinary creativity, sustainability, diet management, allergy mitigation, and reducing food wastage. The computational gastronomy resources present a vast array of data points spanning an extensive resource of recipes from global cuisines (RecipeDB^7^), flavor compounds from natural ingredients (FlavorDB^8^), nutritional correlates of recipes, empirical evidence for food-disease associations (SpiceRx^9^, DietRx^11^), and estimated carbon footprints of recipes (SustainableFoodDB^10^). Application of natural language processing techniques, such as NER^17,27^, and extensive food-related data resources have enabled the creation of novel recipe generation algorithms.

The increasingly sophisticated models for text generation and highly structured recipe data availability have opened up exciting possibilities for imitating culinary creativity with artificial intelligence. We implemented text generation strategies rooted in Large Language Models (LLMs) to build algorithms for creating novel recipes that are not just palatable but are tasty and nutritious. Mining palatable recipes from a galaxy of theoretically possible dish preparations would be a significant step in artificial intelligence toward automating culinary creativity. Fine-tuning open-source LLMs (such as LLaMa^66^ and GPT2^67^) with structured compilations of named entities from recipes enables the generation of recipes of desired culinary style, nutritional profile, and ingredient choices. It can also be leveraged to minimize the price and ecological impact of the recipe. Fine-tuned with GPT2 LLM, Ratatouille^21^ is an algorithm for generating novel recipes. Trained with a structured corpus of over 118,000 recipes richly annotated with state-of-the-art NER algorithms, Ratatouille captures the cumulative culinary intuition accumulated over millennia by cultures across the globe.

In other similar attempts, Cook-Gen^57^ generated cooking actions from recipes to overcome issues with irregular data patterns. FIRE^58^ is a multimodal methodology for recipe generation to create the food title, ingredients list, and cooking instructions by taking food images as input. Another study^55^ focused on enhancing user culinary experiences by leveraging contextual and relational information to rank plausible substitutions to showcase the potential for personalized cooking. In contrast with traditional monolithic approaches, a more agile method^54^ employed image models and multiple data points to address the limitations of existing multimodal models. Along with these data-driven approaches, the generative grammar of cooking ponders on the underlying culinary rules and grammar that dictate the art of cooking^25^.

## The Turing test for chefs

In 1950, Alan Turing, regarded as the father of computer science and artificial intelligence, asked, ‘Can machines think?’^68^ This question has been debated for decades, with some believing in computers’ ability to imitate human intelligence and creativity beyond doubt, while others stubbornly refute such a possibility. Seven decades later, as we explore culinary creativity, we wonder, ‘Can machines think like chefs?’ Can they create novel recipes? Can computer-generated recipe instructions fool a chef into thinking that they are authentic? With breathtaking progress in language generation models, we stand at a point in history where we can conduct a ‘Turing Test for Chef’ that builds on Alan Turing’s inquiry into human intelligence and machines’ ability to imitate.

The ‘Turing Test for Chef’ framework is intended for chefs who will evaluate the efficacy of recipe generation algorithms (Fig. 1b). After a chef enrolls on the platform, they are presented with a randomly selected recipe from the stack of authentic recipes or those created by a text generation model. While the interface has a high granularity to record the chef’s response on a scale of 0 to 5 (0 represents a fake recipe, and 5 stands for an authentic one), the responses were binarized to create a confusion matrix (Fig. 1c). A total of 24 chefs participated in the experiment and evaluated 1,472 recipes, leaving out 30 that chefs skipped. The confusion matrix reflects the true positives (real recipes assessed to be real), true negatives (fake recipes assessed to be fake), false negatives (real recipes assessed to be fake), and false positives (fake recipes assessed to be real) (Fig. 1c). The last class of recipes (false positives) is of special interest to us as they reflect the extent to which the computer-generated recipes resembled the real ones in the chef’s eyes. With an F1 score of 69.88%, our study demonstrates the capabilities of fine-tuned models trained with well-annotated, structured data to generate meaningful recipe texts that can fool chefs, thereby (barely) passing the ‘Turing Test for Chef.’ Of course, the models have much to improve upon in capturing subtle culinary nuances and will improve with reinforcement learning, enhanced training data, and superior model architectures. There is ample scope for training models to include relevant culinary details, including multimodal sensory data such as color and texture, and cooking instruction details unaccounted thus far.

Towards addressing the audacious query, ‘Can computers generate palatable and, hopefully, tasty recipes?’ we show that neural network models can synthesize potentially palatable recipes by implementing a range of text generation models fine-tuned with a gold-standard repository of recipe compositions. While language models have the potential of generating syntactically and semantically coherent text, their text generation abilities with multi-sensorial consequences for taste and odor remain untested hitherto. We are conducting subsequent controlled studies to assess the palatability of such recipes by evaluating cooked dishes through the sensory expert panel. These recipe generation models open a new realm of possibilities to generate palatable recipes by accounting for the constraints of culinary style, ingredient preferences, and allergies. They can potentially be optimized for cost, calories, and carbon footprints. Thus, going beyond the boundaries of creative writing and art, we argue that generative algorithms can help create potentially tasty and nutritious recipes and shape the future of culinary creativity.

## Feeding 10 billion

Human civilization faces the challenge of sustainably feeding an anticipated population of 10 billion^69^. The food system is a crucial contributor to global greenhouse gas emissions central to climate change and global warming. Sustainably feeding billions requires overhauling the food system from farm to fork. The world is looking at systemic interventions to mitigate this crisis, including local and seasonal recipes, alternative protein sources, and plant-based meat. Making aspects of the food system computable will go a long way in these endeavors. Besides fueling culinary creativity, AI-driven recipe generation can be used to address challenges on the frontiers of public health, nutrition, and sustainability.

Breaking down recipes into their ingredients and further down to the molecular level, touching on aspects of flavor and nutrition, opens up exciting opportunities: culinary fingerprints of cuisines, food pairing analysis, and strategies for creating fusion recipes. Knowing the culinary signatures that typify global cuisines is critical, as they are cultural lock-ins that dictate our dietary patterns. The following are among the key questions aligned with this thought: Are there patterns characterizing the nuances of cooking in various cuisines? What culinary features define the uniqueness of a cuisine? How to create personalized recipes? Answers to these questions can help design generative algorithms to design novel recipes of desirable culinary patterns. Further, quantification and structured collation of data also enables optimizing the palatable recipes for their nutritional profiles, prices, and carbon footprints, other than designing recipes suited for lifestyle constraints such as allergy and diet-linked disorders.

## The data-driven future of food

Computational gastronomy presents a data-driven approach to food and paves the way to revolutionizing the food landscape. This new data science will transform our artistic outlook toward food and cooking. Integrating food, data, and computation can transform the culinary landscape with data-driven innovations. Just as the digitization of photographs brought about innovations in hardware and software, introducing a ‘recipe data structure’ will open new avenues for food and cooking. When combined with robotics, such formalism can automate processes and accelerate kitchen innovations. Anything is possible, from a robotic beverage-making machine that will pour tea, coffee, cocktails, or mocktails personalized to match the taste buds to a *biryani*-maker. Understanding the molecular correlates of taste and odor of dishes, ingredients, and their molecules can help inch towards a flavor printer that generates molecular concoctions of desirable sensory profiles. Knowing the precise nutritional correlates of food and an empirical understanding of their health consequences can help create a digital diet coach that recommends personalized diets that suit one’s health parameters. Net-zero recipes will be feasible starting from fine-grained data of the carbon footprints of ingredients through a traceable, block-chain-enabled supply chain. Computational gastronomy presents a new data-driven paradigm with endless possibilities and a bright food future.
